# RAD6 Positively Affects Tumorigenesis of Esophageal Squamous Cell Carcinoma by Regulating Histone Ubiquitination of CCNB1

**DOI:** 10.1186/s12575-022-00165-z

**Published:** 2022-03-23

**Authors:** Yu Deng, Yujiang Li, Tiantong Wu, Xuyuan Chen, Xiang Li, Kaican Cai, Xu Wu

**Affiliations:** 1 Department of Thoracic Surgery, Nanfang Hospital of Southern Medical University, Guangzhou, China; 2 Department of Thoracic and Cardiovascular Surgery, Affiliated Dongguan People’s Hospital, Southern Medical University, Dongguan, China; 3 Department of General Surgery, Nanfang Hospital of Southern Medical University, Guangzhou, China; 4 Department of Emergency Medicine, Nanfang Hospital of Southern Medical University, Guangzhou, China

**Keywords:** Esophageal squamous cell carcinoma, RAD6A, RAD6B, CCNB1, Ubiquitin

## Abstract

Objective Esophageal carcinoma (ESCA) is deadly cancer worldwide with unknown etiology. This study aimed to investigate the impact and mechanism of RAD6 on the development of Esophageal squamous cell carcinoma (ESCC).

Expressions of RAD6A and RAD6B in ESCA were investigated from TCGA dataset and their expressions in tissue sample of ESCA patients and cells were determined. Functional experiments were conducted to explore the impact of RAD6A and RAD6B on malignant characteristics of several kinds of ESCC cells. Animal experiment was established and injected with RAD6A and RAD6B shRNA to evaluate the effect on tumor growth.

RAD6A and RAD6B were up-regulated in ESCC cells and tissues. Overexpressed RAD6A and RAD6B similarly increased ESCC cell proliferation, invasion and migration and silencing of RAD6 exerted opposite effects. Knockdown of RAD6A suppressed tumor growth and decreased the level of H2B, as data demonstrated positive correlation between RAD6A and CCNB1 in ESCC tissues.

Collectively, this study elucidates that RAD6 is up-regulated in ESCC and promotes the progression of ESCC through up-regulation of CCNB1 to enhance H2B ubiquitination. These evidence provide a novel insight into the pathogenesis of ESCC and might contribute to the development of targeted therapy.

## Introduction

Esophageal carcinoma (ESCA) is one of the most common cancers worldwide, responsible for numerous deaths every year [1, 2]. The incidence was higher among men than among women [3, 4]. ESCC is histologically the most prevalent type of Esophageal carcinoma [5]. It is characterized by high mortality since the disorder is usually diagnostic in an advanced stage and it tends to metastasize [6, 7]. Therefore, it is essential to carry out the researches to explore new biomarkers for ESCC. Nowadays, investigators have made great progress in high-throughput sequencing, a supervised method to obtain gene expression data profiles [8–10]. Key ESCC markers could be identified from a large number of patient samples using high-throughput sequencing, which enhances the diagnosis and treatment of ESCC [11–13].

The ubiquitin system plays a central role in diverse biological activties, such as the protein degradation, cell cycle and apoptosis [14, 15]. The system mainly is composed of three types of enzymes, the ubiquitin-activating enzyme, the ubiquitin-conjugating enzyme, and the ubiquitin ligase enzyme [14, 16]. RAD6 is a kind of ubiquitin-conjugating enzyme found in yeast and it is involved in the DNA repair process [17]. In recent years, previous studies have reported that RAD6 is associated with the development and metastasis of various tumors, including melanoma [18, 19], ovarian cancer [20] and breast cancer [21]. Human RAD6 is encoded by two genes, RAD6A and RAD6B. The amino acid sequences encoded by the two genes are highly homologous, and functionally similar [22]. Abnormal expression of RAD6A has been widely reported in diverse neurological diseases. It is noted that RAD6A expression is significantly up-regulated in the dorsolateral prefrontal cortex of patients with depression, suggesting alterations in chromatin conformation [23]. As a part of the 26S proteasome, RAD6A participates in the clearance of amyloid peptides by protein degradation to reduce accumulation of amyloid protein [24]. Additionally, highly expressed RAD6A is noted to correlate with the poor prognosis of hepatocellular carcinoma (HCC), indicating that RAD6A may be involved in the pathogenesis of HCC [25]. RAD6B also has diverse physiologic functions in biological processes, such as meiosis and tumor formation. The blockade of RAD6B leads to the destruction of meiosis and induces apoptosis during spermatogenesis, causing oligospermia [26]. In melanoma, knockout of RAD6B restrains the development of the condition through Wnt/β-catenin signaling [27]. Clinical data have demonstrated that overexpression of RAD6B decreases the sensitivity of rectal cancer cells to ionizing radiation, suggesting that RAD6B is a poor prognostic factor for rectal cancer patients after radiotherapy [28].

Histones are highly basic proteins that bind to the DNA to form chromatin. Recent studies have shown that histone ubiquitination plays an important role in regulating gene transcription. Accumulating evidence has depicted that E2 enzyme modifies histone ubiquitination. As an E2 enzyme, HR6B interacts with E3 enzyme UBR2 and histone H2A, transporting ubiquitin to H2A to regulate gene expression [29]. RAD6B also facilitates the ubiquitination of H2A and H2B and contributes to chromatin remodeling [30].

Cyclin B1 protein, a member of the cyclin family, is encoded by the CCNB1 gene, and governs normal G2/M phase progression [31, 32]. Cyclin B1 is involved in the progression of many cancers. In HCC, RBM43 binds to the 3'UTR of mRNA to inhibit cyclin B1 expression, thereby suppressing the development of HCC [33]. BMAL1 decreases the proliferation of U87MG glioblastoma cells through down-regulation of cyclin B1 [34]. Previous studies have depicted the relationship between RAD6 and cyclin, as RAD6 enhances H2B ubiquitination to up-regulate the expression of cyclin D1 [35]. However, the interaction between RAD6 and cyclin B1 remains unclear.

The data downloaded from TCGA database demonstrate that RAD6A and RAD6B may play a role in the development of ESCC. However, little is known the functions of RAD6A and RAD6B in ESCC. Therefore, this study established animal model of ESCC and examined the expression of RAD6A and RAD6B in ESCC cells and tissues to identify their impacts on the condition.

## Materials and Methods

### Bioinformatics Analysis

This study used Ualcan (http://ualcan.path.uab.edu/index.html) to extract the gene expression levels and prognosis of RAD6A and RAD6B from TCGA-ESCA data.

### Patient Samples

Clinical specimens and adjacent normal tissues were collected from 50 ESCA patients who underwent surgery in Nanfang Hospital of Southern Medical University from 2019 to 2020. This experiment has obtained informed consent from each patients and approval of the Ethics Committee. The tissues were stored at -80 °C immediately for subsequent qRT-PCR analysis.

### Cell Culture

Six kinds of ESCC cells (SHEE, KYSE180, TE8, KYSE150, KYSE410 and KYSE520) were purchased from ATCC and cultured in RPMI 1640 medium with 10% fetal bovine serum and 1% antibiotics at 37 °C in 5% CO_2_.

### qRT-PCR

Total RNA was extracted using TRIzol reagent (Invitrogen, Shanghai, China), followed by transcription into cDNA with Reverse Transcription System (Promega, Beijing, China). The cDNA was subjected to amplification using SYBR Green qPCR SuperMix (Invitrogen, Shanghai, China) and ABI PRISM® 7500 Sequence Detection System (Applied Biosciences, California, USA). The primers used for qPCR are listed in Table 1.

**Table 1:** Sequence of primers for real-time PCR

Primer	Forward Sequence (5’ to 3’)	Reverse Sequence (5’ to 3’)
GAPDH	ACGGATTTGGTCGTATTGGG	TGATTTTGGAGGGATCTCGC
RAD6A	GGATGGAACATTTAAACTTAC	TGCTGGACTATTGGGATTG
RAD6B	GCTGGATGAACCGAATCCTA	TTCAACAATGGCCGAAACTC
CCNB1	AACTTTCGCCTGAGCCTATTTT	TTGGTCTGACTGCTTGCTCTT

### Immunohistochemical Analysis

As previously described, immunohistochemistry was conducted to examine protein expression in tissues [36]. Briefly, ESCC tissues and adjacent normal tissues were cut into 3 μm paraffin sections and incubated with mouse monoclonal antibodies RAD6 (1:200, Abcam, USA) at 4 °C overnight. The sections were probed with goat anti-mouse IgG-HRP secondary antibodies (1:2000, Southern Biotech, USA) for 1.5 h at room temperature and stained with DAB reagent, and finally counterstained with hematoxylin. The intensity was scored as follows: 0, negative; 1, weak; 2, moderate and 3, strong. The frequency of positive eckks was defined as follows: 0, less than 5%; 1, 5% to 25%; 2, 26% to 50%; 3, 51–75%; 4, more than 75%.

### Cell Transfection and Lentivirus Packaging

Human-derived RAD6A and RAD6B and control plasmids acquired from addgene were cloned into the pLVX-IRES-Puro-3 × Flag vector. The lentivirus (Hanheng Biotechnology, Shanghai, China) was loaded with shRNA targeting RAD6A and RAD6B and the corresponding controls (Sigma-Aldrich, Shanghai, China). KYSE180 and TE8 cells were then infected with the packaged lentivirus and stable expression strains were screened with 5 μg/ml puromycin. The target sequences of shRNA are listed in Table 2.

**Table 2:** Target sequence of shRNA

Name	Sequence (5’ to 3’)
shNC	CAGAAGGGGCGGAGATGAT
shRAD6A#1	TGGTGTGGAACGCGGTCATTT
shRAD6A#2	GTCTATGCAGATGGTAGTATA
shRAD6B#1	CGGGATTTCAAGCGGTTACAA
shRAD6B#2	GAATCCTAACAGTCCAGCCAA

### Western Blot Analysis

Cells were lysed in RIPA Lysis Buffer (Sigma-Aldrich, Shanghai, China) with proteinase and phosphatase inhibitors. With protein quantification using a BCA assay kit (KeyGEN Biotech, Jiangsu, China), protein extractions (50 μg) were seeded on SDS-PAGE, then transferred to PVDF membrane (Sigma-Aldrich, Shanghai, China). The membrane was incubated with the corresponding primary antibody at 4 °C overnight and the HRP-labeled secondary antibodies for 2 h for visualization analysis. Antibodies information are listed in Table 3.

**Table 3:** Antibody

Name	Supplier	Catalog
GAPDH	Aksomics	KC-5G5 (1: 10,000)
Flag	Abcam	Ab1162 (1: 5000)
RAD6	Abcam	ab31917 (1: 5000)
CDK1	Abcam	ab133327 (1: 10,000)
Cyclin A	Abcam	ab53699 (1: 1000)
Cyclin B	Abcam	ab32053 (1: 1000)
Rabbit Anti-Mouse IgG H&L	southern biotech	6170–05 (1: 10,000)
Goat Anti-Rabbit IgG H&L	southern biotech	4050–05 (1: 20,000)

### CCK-8 Assay

KYSE180 and TE8 cells (1 × 10^5^ cells/ml) were plated in a 96-well plate. After adherence to the wall, 10 μl of CCK8 detection solution was added to each well and incubated for 4 h. Measurements were taken at different time periods (0 h, 24 h, 48 h, 72 h) by placing the sample under the Multiscan MK3 microplate reader (ThermoFisher, Shanghai, China).

### Transwell Assay

KYSE180 and TE8 cells (1 × 10^5^ cells) were resuspended in serum-free medium, and transferred to the upper chamber of the cell culture plate, and complete medium was added to the lower chamber. After 48 h of incubation, the chamber was taken out. The cells in the upper chamber were wiped off, fixed with 4% paraformaldehyde, washed with PBS and stained with crystal violet for 10 min. For invasion detection, cell suspension without FBS was plated on the the upper chamber of Transwell coated with diluted Matrigel, and 600 μl of complete medium was added to the lower chamber. The cells were stained after incubation, and photographed for analysis.

### Xenograft Mouse Model

All animal experiment procedures were performed in accordance with the guidelines approved by the Ethics Committee. Nude mice (5 weeks old, 20 ± 2.2 g) were purchased from Shanghai Laboratory Animal Center. RAD6A shRNA-treated TE8 cells at the logarithmic growth phase were subcutaneously injected (1 × 10^6^ cells) into the flanks of nude mice. The tumor volume was calculated by measuring the length (L) and width (W) every 7 days. After 5 weeks, the mice were sacrificed, and the tumor tissues were taken out for HE staining analysis.

### Chromatin Immunoprecipitation (ChIP)

As previously described [20], ChIP was carried out to determine the content of H2B protein. Briefly, TE8 cells transfected with RAD6A shRNA or control were cultured and sonicated to separate the chromatin for ChIP. The cells were probed with H2B antibody or rabbit IgG overnight. With adsorption of the chromatin containing H2B protein, the antibody-protein conjugate was digested with proteinase K, and then the product was recovered for subsequent PCR analysis. For detection of CCNB1 site, the following primer set was used: 5′-TCAGTTCCTCCAACCCAGAGA-3′ and 5′-GCCACAGTCTCAGAAGTGTCA-3′.

### Statistical Analysis

Data were analysed by the SPSS v23.0 software (SPSS Inc., Chicago, USA). Chi-square test detected the correlation between RAD6 and CCNB1, and the difference in 2 groups or multiple groups were analysed by student t-test or one way analysis of variance (ANOVA). *P* < 0.05 indicates statistical significance.

## Results

### High Expression of RAD6A and RAD6B in Esophageal Carcinoma Indicates Poor Prognosis

**Fig. 1:**
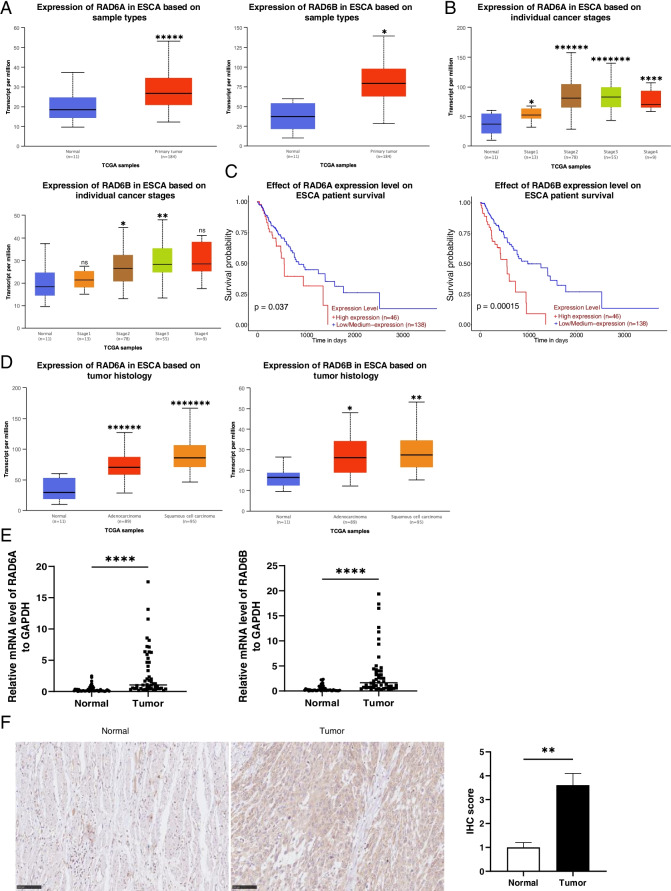
RAD6A and RAD6B are highly expressed in EC. **A** RAD6A and RAD6B expression were evaluated in TCGA database; **B** Expression of RAD6A and RAD6B in different tumor stages; **C:** Survival analysis for RAD6A and RAD6B in ESCA; **D** Expression of RAD6A and RAD6B in different histological types; **E** mRNA expression of RAD6A and RAD6B were evaluated in 50 ESCC patients; **F** Representative pictures of RAD6 staining in ESCC patients. *** *P* < 0.001, **** *P* < 0.0001, vs Normal

TCGA data showed that in the advanced stage of Esophageal carcinoma, the expression of RAD6A and RAD6B are highly expressed while the survival rate are decreased, which suggests poor prognosis (Fig. 1A, B and C). In addition, histological type results showed that the expression of RAD6A and RAD6B was higher in squamous cell carcinoma than in adenocarcinoma (Fig. 1D). To confirm the up-regulation of RAD6A and RAD6B, ESCC tissues and adjacent normal tissues were collected from patients with ESCC (*n* = 50), and examined by qRT-PCR and immunohistochemistry analyses (Fig. 1E and F). The results consistently confirmed the increased expression of RAD6A and RAD6B in ESCC tissues relative to normal tissues. Collectively, these data indicate that RAD6A and RAD6B may be involved in the development of ESCC.

### Overexpression of RAD6 Increases ESCC Cell Proliferation, Migration and Invasion

**Fig. 2:**
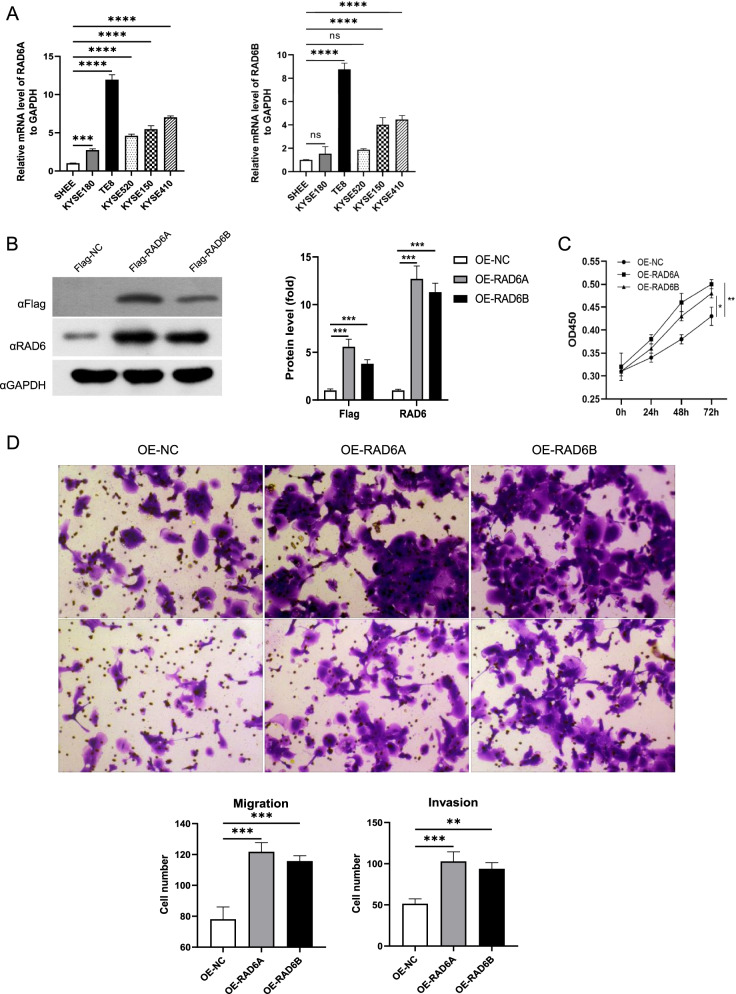
Overexpression of RAD6A and RAD6B promote cell proliferation, migration and invasion of KYSE180 cells in vitro. **A** mRNA expression of RAD6A and RAD6B were evaluated in ESCC cells. **B-D** KYSE180 cells were infected with lentiviruses expressing RAD6A or RAD6B or control. **B** Western blot analysis of RAD6; **C** CCK-8 assay was used to assess the effect of cell proliferation after overexpressing RAD6A and RAD6B; **D** and **E** Transwell assays were used to evaluate the effect of cell migration and invasion after overexpressing RAD6A and RAD6B. ** *P* < 0.01, *** *P* < 0.001, **** *P* < 0.0001, vs OE-NC

To further examine the effects of RAD6A and RAD6B on the progression of ESCC at the cellular level, we examined their expressions in several ESCC cell lines using qRT-PCR analysis. The results showed that the expressions of RAD6A and RAD6B were increased in all ESCC cell lines with TE8 cells exhibiting highest level and KYSE180 exhibiting lowest level (Fig. 2A). Based on their expression levels, KYSE180 cells were chosen to established the overexpressed cell model by infecting lentivirus loaded with Flag-RAD6A and Flag-RAD6B plasmids. Western blot results confirmed the up-regulation of RAD6A and RAD6B in cells upon infection (Fig. 2B). Importantly, the functional experiments revealed that overexpression of RAD6A and RAD6B increased the proliferation, migration and invasion of KYSE180 cells (Fig. 2C and D). These results suggest that overexpression of RAD6A and RAD6B enhances the ESCC cell proliferation, migration and invasion.

### Knockdown of RAD6A/RAD6B Decreases the Proliferation, Migration and Invasion of ESCC Cells

**Fig. 3:**
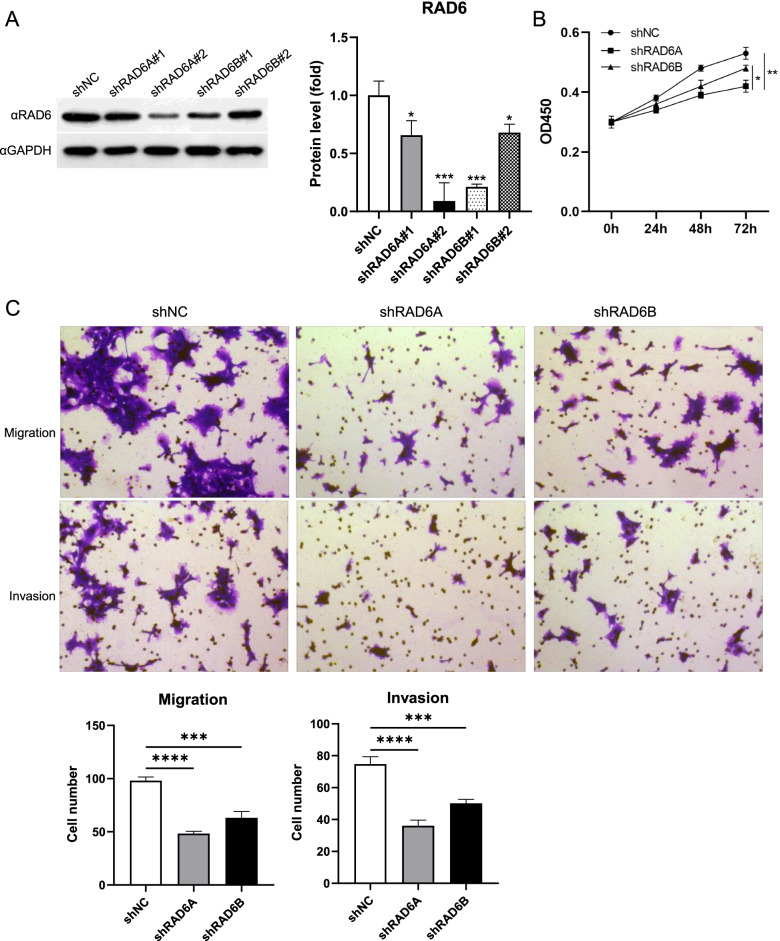
Knockdown RAD6A and RAD6B inhibit cell proliferation, migration and invasion of TE8 cells in vitro. TE8 cells were infected with lentiviruses expressing shRNA targeting RAD6A or RAD6B or shRNA control. **A** Western blot analysis of RAD6; **B** CCK-8 assays was used to assess the effect of cell proliferation after knocking down RAD6A and RAD6B; **C** and **D** Transwell assays were used to evaluate the effect of cell migration and invasion after knocking down RAD6A and RAD6B. * *P* < 0.05, *** *P* < 0.001, **** *P* < 0.0001, vs sh-NC

Next, a stably knockdown cell line was constructed in TE8 cells using shRNA. Two shRNA sequences to target RAD6A and RAD6B were designed, respectively, and shRAD6A#2 and shRAD6B#1 exhibited greater knockdown efficiency, as demonstrated by Western blot detection (Fig. 3A). It was further found that silencing of RAD6A and RAD6B decreased the proliferation, migration and invasion of TE8 cells (Fig. 3B and C). Overexpression and knockdown results suggest that both RAD6A and RAD6B enhance the malignant characteristics of ESCC cells at the cellular level, but RAD6A exerts a more significant effect, although this may be related to the efficiency of the plasmids.

### Knockdown of RAD6A/RAD6B Inhibits Tumor Formation in Mice

**Fig. 4:**
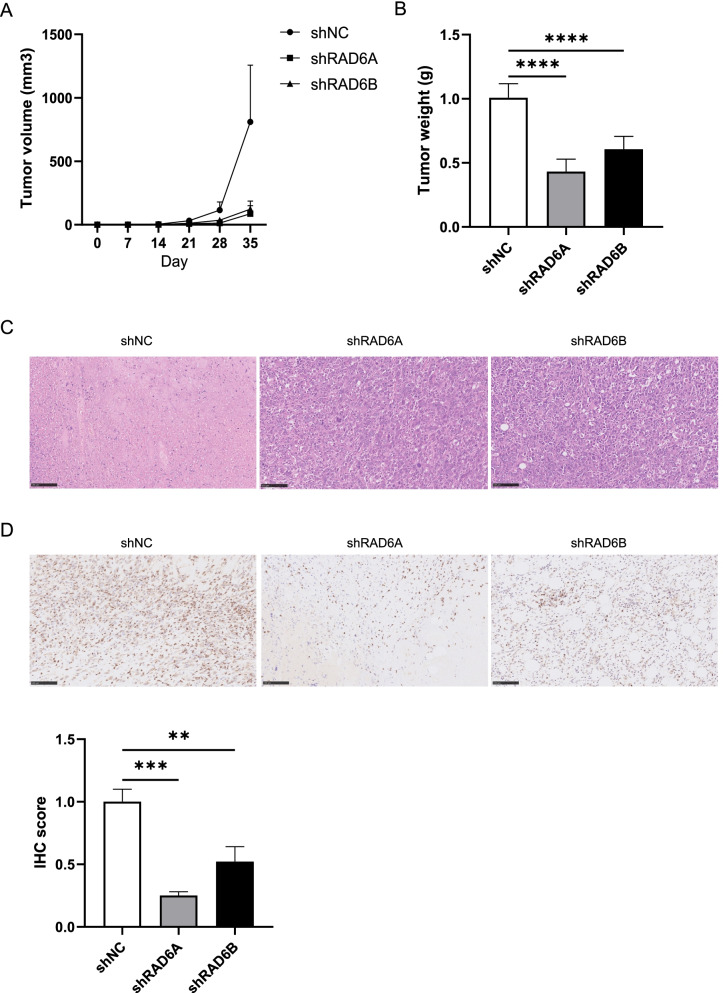
Suppression of RAD6A expression inhibits ESCC growth in vivo. TE8 cells with or without depletion of RAD6A were injected into nude mice. **A** Volumes of xenograft tumors were measured and plotted; **B** weights of xenograft tumors; **C** Representative HE staining in tumor; **D** Representative immunohistochemical detection of RAD6A in tumor. ** *P* < 0.01, *** *P* < 0.001, **** *P* < 0.0001, vs sh-NC

Subsequently, in vivo experiments were performed to verify the functions of RAD6A and RAD6B. After identification of RAD6A/RAD6B’s impact on ESCC cells, TE8 cells infected with shRAD6A#2 and shRAD6B#1 were injected into the dorsal side of the nude mice. The tumor volume was measured every 7 days, and the mice were sacrificed 35 days later. The data demonstrated that compared with the control group, RAD6A knockdown resulted in a decrease in the tumor (Fig. 4A and B). Then HE staining results showed that tumor formation slowed down and histological structure were more normal after RAD6 knockdown (Fig. 4C). Immunohistochemical analysis confirmed the decreased expression of RAD6A in the transplanted tumors of the knockdown RAD6A group (Fig. 4D). These results prove that RAD6 knockdown suppresses tumor growth in ESCC.

### RAD6A Promotes CCNB1 Transcription Through H2B Monoubiquitin

**Fig. 5:**
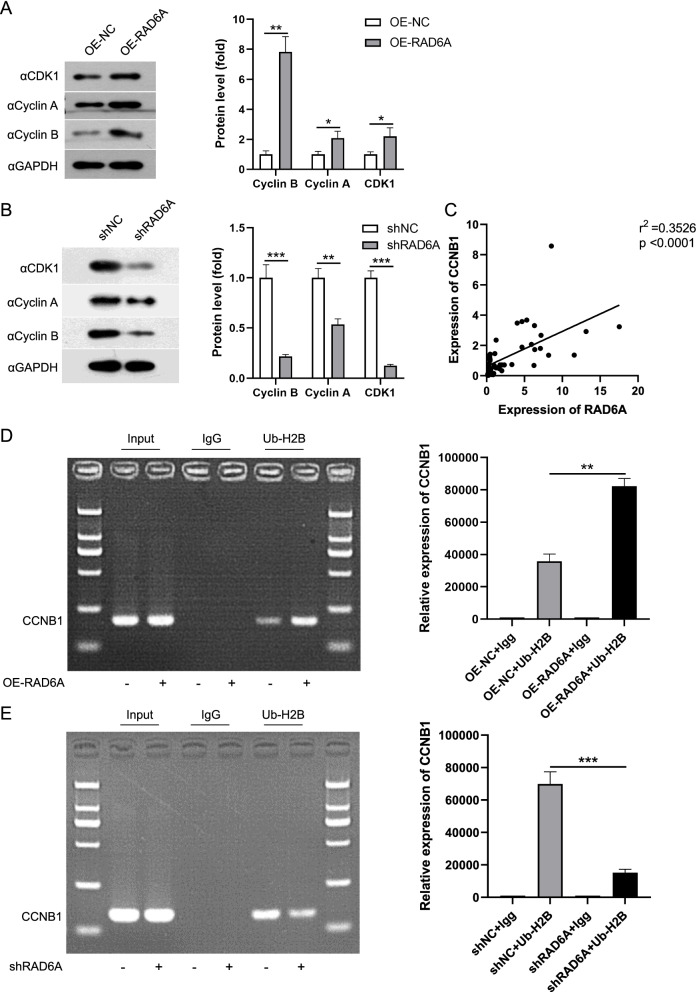
RAD6A regulates CCNB1 through pro-transcription histone modifications. **A** and **B** Western blot analysis of cell cycle-related proteins; **C** qRT-PCR was used to detect the correlation between RAD6A and CCNB1 expression in ESCC patients; **D** and **E** ChIP was used to determine if suppression of RAD6A altered the level of pro-transcription H2B-Ub in the promoters of CCNB1 genes. ** *P* < 0.01, *** *P* < 0.001, vs sh-NC

Previous studies have shown that several cell cycle related proteins are involved in cell proliferation, such as CCNB1 [37]. To investigate potential RAD6A targets, we checked up the expression of cell cycle-related factors in KYSE180 cells and TE8 cells. Overexpression of RAD6A induced up-regulation of CDK1, Cyclin A and Cyclin B, and especially expression of Cyclin B declined most significantly (Fig. 5A). Silencing RAD6A inhibited the expression of CDK1, Cyclin A and Cyclin B (Fig. 5B). Then the mRNA expression of CCNB1 in the tissue samples of patient with ESCC. The results showed that the expression of RAD6A was positively correlated to CCNB1 expression in ESCC tissues (Fig. 5C).

It has been reported that RAD6 regulates downstream methylation through histone H2B modification, and modifies gene transcription [38]. To identify whether RAD6A stimulates CCNB1 gene expression through chromatin modification, CHIP analysis was performed in KYSE180 cells and TE8 cells. The results showed that overexpression of RAD6A promoted H2B ubiquitination on CCNB1 promoter (Fig. 5D). After knockdown of RAD6A, the level of H2B-Ub on the CCNB1 promoter was significantly lower than that of the control group (Fig. 5E). These results suggest that RAD6A can regulate the expression of CCNB1, which may be the reason for its involvement in regulating ESCC cell proliferation.

## Discussion

The RAD6 protein has been reported to participate in the development of various cancers where it mainly acts as an oncogene. In ovarian cancer (OC), RAD6 is noted to induce chemical resistance and potentiate the DNA repair ability of OC cells [20]. It modifies the pathogenesis and development of melanoma and breast cancer through the Wnt/β-catenin pathway [27, 39]. Interestingly, in patients with advanced chronic myeloid leukemia, abnormal expression of RAD6 leads to decreased activity and ultimately damages bone marrow differentiation and triggers blast crisis [40]. The function of RAD6 in ESCC has not been reported.

This study found that RAD6A and RAD6B are highly expressed in ESCC tissues and suggests poor prognosis. Functional experiments demonstrated RAD6A and RAD6B promoted the proliferation, migration and invasion of ESCC cells. Xenograft tumor experiments verified that knockdown of RAD6A restrained tumorigenesis in mice. The chip experiment verified that RAD6A regulated the ubiquitination of H2B, thereby regulating the expression of cyclin B1.

In most previous studies, investigators tended to directly focus on the RAD6 protein instead of distinguishing between RAD6A and RAD6B for separate investigation. In fact, the functions of RAD6A and RAD6B are not completely identical in different diseases. The expression of RAD6B in metastatic melanoma is greater than that of RAD6A, and there are cases of co-expression of wild-type and mutant genes [19]. Besides, RAD6A deficiency is associated with neurological diseases [24], while RAD6B deficiency drives oligospermia and infertility [26]. These studies have shown that although both RAD6A and RAD6B encode RAD6, there may be slight differences in the molecular mechanisms underlying their roles in diseases. The results of this study noted that up-regulation or down-regulation of RAD6A exerts a more significant impact on the proliferation, migration and invasion of ESCC cells than RAD6B. This result may be partly due to the knockdown efficiency or overexpression efficiency, or due to the higher content of RAD6A mRNA in ESCC cells.

Accumulating evidence has reported that RAD6 regulates the transcription of multiple genes through histone H2A or H2B ubiquitination [20, 30, 35, 38]. The genes regulated by RAD6 also include multiple cell cycle-related proteins. RAD6 increases H2B ubiquitination and H3K4me3 levels in the CCND1 promoter region, thereby up-regulating the expression of cyclin D1 [35]. Another paper reveals that knockdown of CDK9 and RAD6A induces a decrease in H2B ubiquitination [41]. Importantly, our work elucidates that RAD6A affects CCNB1 expression by regulating H2B ubiquitination on the CCNB1 promoter, which might be a potential pathway for RAD6 to regulate ESCC cell proliferation.

In conclusion, the current study confirms that RAD6 promotes the progression of ESCC, indicating that RAD6 may be a new biomarker for molecular diagnosis and prognosis of cancer therapy.

## Data Availability

All data generated or analyzed during this study are included in this published article.

## Abbreviations

ESCC: Esophageal squamous cell carcinoma
HCC: Hepatocellular carcinoma
CCK8: Cell counting kit-8
ChIP: Chromatin immunoprecipitation
OC: Ovarian cancer
